# Living in the Past: Phylogeography and Population Histories of Indo-Pacific Wrasses (Genus *Halichoeres*) in Shallow Lagoons versus Outer Reef Slopes

**DOI:** 10.1371/journal.pone.0038042

**Published:** 2012-06-06

**Authors:** William B. Ludt, Moisés A. Bernal, Brian W. Bowen, Luiz A. Rocha

**Affiliations:** 1 Department of Marine Science, University of Texas, Austin, Texas, United States of America; 2 Hawaiian Institute of Marine Biology, University of Hawaii, Kaneohe, Hawaii, United States of America; 3 Department of Ichthyology, California Academy of Sciences, San Francisco, California, United States of America; Brigham Young University, United States of America

## Abstract

Sea level fluctuations during glacial cycles affect the distribution of shallow marine biota, exposing the continental shelf on a global scale, and displacing coral reef habitat to steep slopes on oceanic islands. In these circumstances we expect that species inhabiting lagoons should show shallow genetic architecture relative to species inhabiting more stable outer reefs. Here we test this expectation on an ocean-basin scale with four wrasses (genus *Halichoeres*): *H. claudia* (N = 194, with ocean-wide distribution) and *H. ornatissimus* (N = 346, a Hawaiian endemic) inhabit seaward reef slopes, whereas *H. trimaculatus* (N = 239) and *H. margaritaceus* (N = 118) inhabit lagoons and shallow habitats throughout the Pacific. Two mitochondrial markers (cytochrome oxidase I and control region) were sequenced to resolve population structure and history of each species. Haplotype and nucleotide diversity were similar among all four species. The outer reef species showed significantly less population structure, consistent with longer pelagic larval durations. Mismatch distributions and significant negative Fu’s F values indicate Pleistocene population expansion for all species, and (contrary to expectations) shallower histories in the outer slope species. We conclude that lagoonal wrasses may persist through glacial habitat disruptions, but are restricted to refugia during lower sea level stands. In contrast, outer reef slope species have homogeneous and well-connected populations through their entire ranges regardless of sea level fluctuations. These findings contradict the hypothesis that shallow species are less genetically diverse as a consequence of glacial cycles.

## Introduction

Glacial cycles and associated sea level changes during the Pleistocene have historically affected the distribution of plants and animals in both terrestrial and marine environments [1], [2]. Sea levels dropped as much as 120 m during the last glacial maxima approximately 17,000 years ago, exposing large areas of continental shelf [3], [4], restricting reefs to vertical or steep slopes on oceanic islands and reducing, drastically changing, or eliminating the habitat for shallow water species [5]. During these events, lagoon species may have undergone population bottlenecks or local extirpation [6], while species adapted to the outer reef edges could use deeper reefs as refuges [7]. As a result, population genetic patterns should be closely linked to habitat preference. Fluctuations in habitat area in the Pleistocene due to sea level change are suspected to be a primary influence on population histories in several marine taxa, including marine gastropods [8], crabs [9], fish [10], and corals [11].

Habitat preferences differ markedly among closely related species of wrasses (genus *Halichoeres*; family Labridae). The sister species *H. ornatissimus* and the recently described *H. claudia* are usually found on the outer margins of reefs [12], [13]. *H. claudia* spans the Indo-Pacific, from Cocos-Keeling in the eastern Indian Ocean, to French Polynesia and the Line Islands in the Central Pacific, and north to Indonesia, whereas *H. ornatissimus* is restricted to the Hawaiian Islands [13]. Two other wrasses of the same genus occupy a range similar to *H. claudia*: *H. trimaculatus* and *H. margaritaceus*. However, these latter two species occupy shallower habitats in lagoons and reef flats. *H. trimaculatus* is found in shallow lagoons and bays among sand, rubble, and small coral heads, as shallow as a few centimeters, and rarely deeper than 10 m [14]. *H. margaritaceus* is commonly found in shallow reefs and rocky shores exposed to wave surge within the top 3 m of the water column [14], [15]. Genetic architecture will also be influenced by contemporary habitat area and population sizes, but these are uniformly vast in these wrasses.

Previous studies have examined genetic diversity in lagoon and outer reef fish, and found that lagoon species have less genetic diversity than outer reef species [6], [16]. In each case these conclusions were based on sampling one or a few locations. However, to completely understand the genetic structure of a species it is preferable to sample the entire range [17]. Therefore the goal of this study was to assess the genetic consequences of sea level changes on lagoon and outer reef fishes sampled across the Pacific.

Here we address two questions in the context of range-wide phylogeography: 1) what is the genetic (mtDNA) diversity of each species across their range, and 2) have historical factors differentially influenced the genetic diversity and population structure of species with different habitat preferences? We hypothesize that sea level lows resulted in population bottlenecks for lagoon species which should cause decreased genetic diversity when compared to outer reef species. To address these questions we used two mtDNA segments, cytochrome oxidase I (CO1) and the control region (CR). These markers were chosen so that our results could be directly compared to previous studies as well as to examine different temporal regimes with the more conservative CO1 and the more rapidly evolving (non-coding) CR segment.

## Methods

### Sampling and Extraction

**Figure 1:**
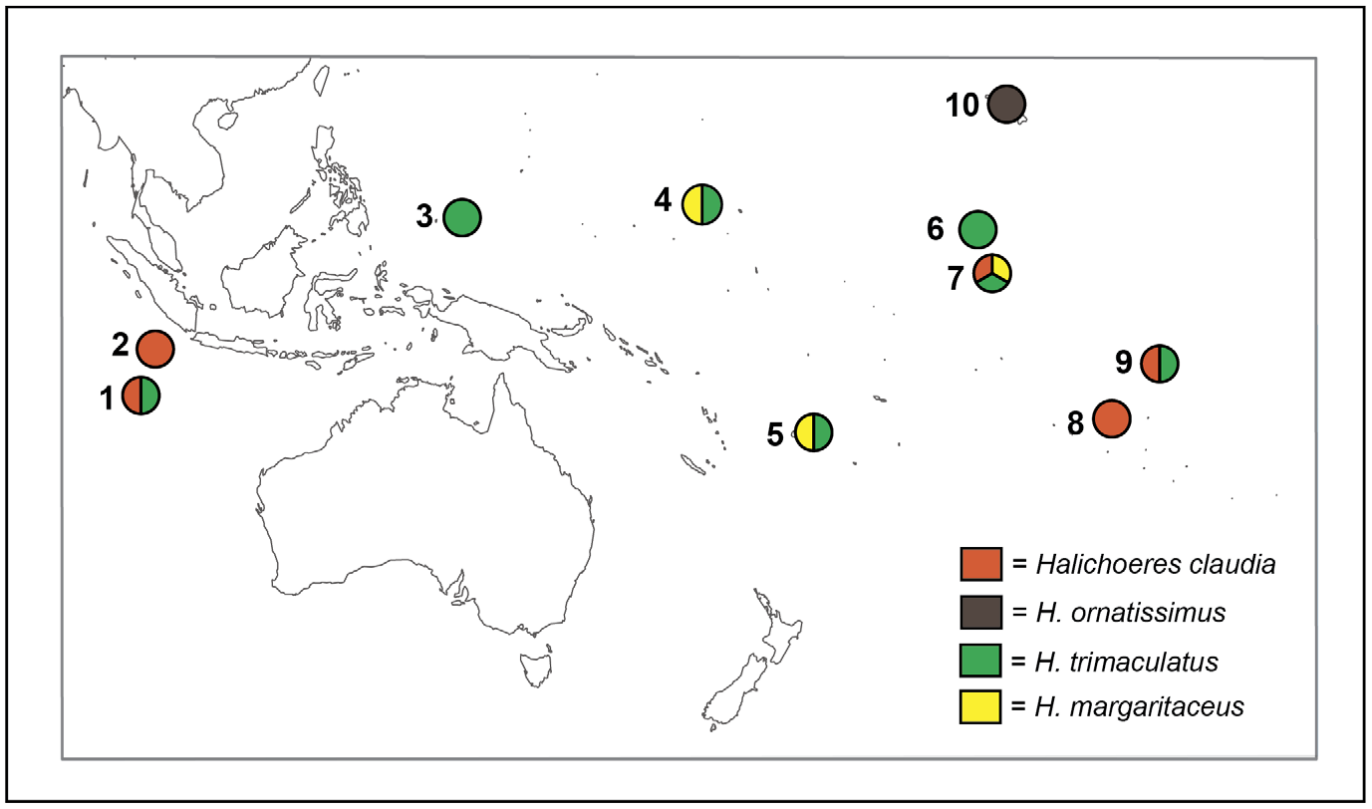
Sampling locations per species. Map of the Indo-Pacific region where samples of all species were collected. 1) Cocos Keeling Island, 2) Christmas Island, 3) Palau, 4) Kwajalein, Marshall Islands, 5) Fiji, 6) Palmyra, 7) Kiribati, 8) Moorea, 9) Marquesas, 10) Hawaiian Archipelago. Colors in the pie charts indicate species sampled at each location.

We sampled *Halichoeres claudia* (n = 194, 5 locations), *H. ornatissimus* (n = 346, 13 locations), *H. trimaculatus* (n = 239, 7 locations), and *H. margaritaceus* (n = 118, 3 locations) from 21 locations in the Indian and Pacific Oceans (Fig. 1). All samples were collected using pole spears while SCUBA diving or snorkeling between 2006 and 2009. Fin clips or gill tissues were subsequently stored in either 95% ethanol or saturated salt (NaCL) solution with 20% DMSO [18]. DNA was later extracted using the “Hot-Shot” method described in Meeker et al. [19] and stored at 10°C before PCR amplification. All necessary permits were obtained for the described field studies. Permits were obtained from the following authorities: US Fish and Wildlife Service, Hawaii Department of Land and Natural Resources, Hawaii Papahānaumokuākea Marine National Monument, Palau Marine Resources Office, Marshall Islands Marine Resource Authority, Kiribati Ministry of Fishes and Marine Resources, Australia Department of Environment and Natural Resources, French Polynesia Fisheries Department, and the Fiji Ministry of Fisheries and Forests.

### Laboratory Procedures

Both CO1 and CR were amplified for all specimens. CO1 segments were amplified with primers BOL-F1 (5′ TCA ACY AAT CAY AAA GAT ATY GGC AC 3′) and BOL-R1 (5′ ACT TCY GGG TGR CCR AAR AAT CA 3′) [20]. Each 25µl reaction was comprised of approximately 10 ng DNA, 3.5 mM MgCl_2_, 1× buffer, 0.18 µM of each primer, 2.5 mM DNTP, and 2 units of GoTaq DNA Polymerase (Promega). Polymerase chain reactions were conducted using a temperature profile of a one minute denaturing step at 95°C, followed by a 30 second annealing temperature of 45–52°C depending on the species, and completed with an extension of 45 seconds at 72°C, for 32 cycles. Samples were then purified and sequenced using the BOL-F1 primer. CR segments were amplified with primers CRA (5′ TTC CAC CTC TAA CTC CCA AAG CTA G 3′) and CRE (5′CCT GAA GTA GGA ACC AGA TG 3′) [21]. Temperature profiles for PCR amplification were similar to CO1 except annealing temperature was 52–55°C depending on the species. PCR amplifications for both loci included aliquots without genomic DNA (negative controls) to detect possible contamination. PCR products were verified with 1.5% agarose gel electrophoresis using GelStart Nucleic Acid Stain (Cambrex Bio Science). Amplicons were then purified and sequenced at the ICMB Core Facilities, University of Texas at Austin, using an ABI 3730 automated sequencer (Applied Biosciences). Samples were sequenced using the forward primer, and rare or questionable haplotypes were sequenced in both directions.

### Population Genetic Analyses

Sequences were aligned using Geneious 5.0.2 (Biomatters). Haplotype and nucleotide diversity (*h* and *π* respectively) [22] were calculated in Arlequin 3.5.1.2 [23]. Pairwise Φ_st_ values were calculated with 1,000 permutations between all sampling locations in Arlequin. Population subdivisions were detected with an analysis of molecular variation (AMOVA) [24], and isolation by distance was analyzed using a Mantel test with 1000 permutations performed in Arlequin. The Mantel test compared straight-line distance between locations (km) to pairwise Φ_st_ values. Migrate 3.1.4 [25] was applied to estimate the overall strength and direction of migration between populations. For each species 10 runs of Migrate were performed, each consisting of 10 short chains of 100,000 steps, and one long chain of 1,000,000 steps, sampling every 100 increments for each. In addition 10 heated chains with varying temperatures (1–10) were used, with a swapping interval set to 1. Migrate runs were then averaged and checked for convergence. Effective population sizes were also estimated using theta values generated in Arlequin following procedures outlined in [26].

### Phylogeographic and Coalescent Analyses

The most appropriate nucleotide substitution models were chosen based on maximum likelihood scores under the AIC approach in Modeltest 3.7 [27]. Coalescent ages were estimated with BEAST 1.6.1 [28] using the most appropriate substitution model with a population expansion model which was verified by mismatch distributions. A strict mutation rate of 3% per million years (MY) between lineages was employed for CO1 [29] and 9% per MY between lineages for CR. The mutation rate for CO1 is based on published literature values for trans-isthmian wrasses which range from 2.3%/MY to 3.4%/MY (Table 3 in [29]). The mutation rate for CR is based on the pairwise divergence of a trans-isthmian pair of damselfish, *Chromis atrilobata* (Accession numbers: EF489847.1- EF489848.1) and *Chromis multilineata* (Accession numbers:EF489843.1-EF489844.1) using the software MEGA 4 [30], assuming 3 million years divergence [29]. CR mutation rates are controversial in fishes, and this rate should be cautiously regarded as a first-order approximation. Each BEAST run was conducted with a chain length of 10,000,000. Runs were repeated until the effective sample size was larger than 200. Multiple runs were then combined using LogCombiner 1.6.1 and then viewed using Tracer 1.5. Bayesian skyline plots were generated using BEAST to estimate the population growth rate for each species through time. Skyline plots ran for 20,000,000 iterations each, with a 10% burn-in for each marker, and were then viewed in Tracer 1.5 to assure the effective sample size was sufficient.

Minimum spanning networks (MSNs) were constructed in TCS 1.21 [31]. For CO1 the connection limit was set to 95%, while for CR a fixed connection limit was set at 50 steps. MSNs were redrawn in Adobe Illustrator CS5 following procedure outlined by Templeton et al. [32].

### Population Expansion and Neutrality

Sequences were checked for neutrality using Fu’s F [33] implemented in Arlequin using 1000 simulated samples. Pairwise mismatch distributions [34] were also constructed comparing observed and expected mismatch distributions under a demographic expansion model in Arlequin.

## Results

### Genetic Diversity

**Figure 2:**
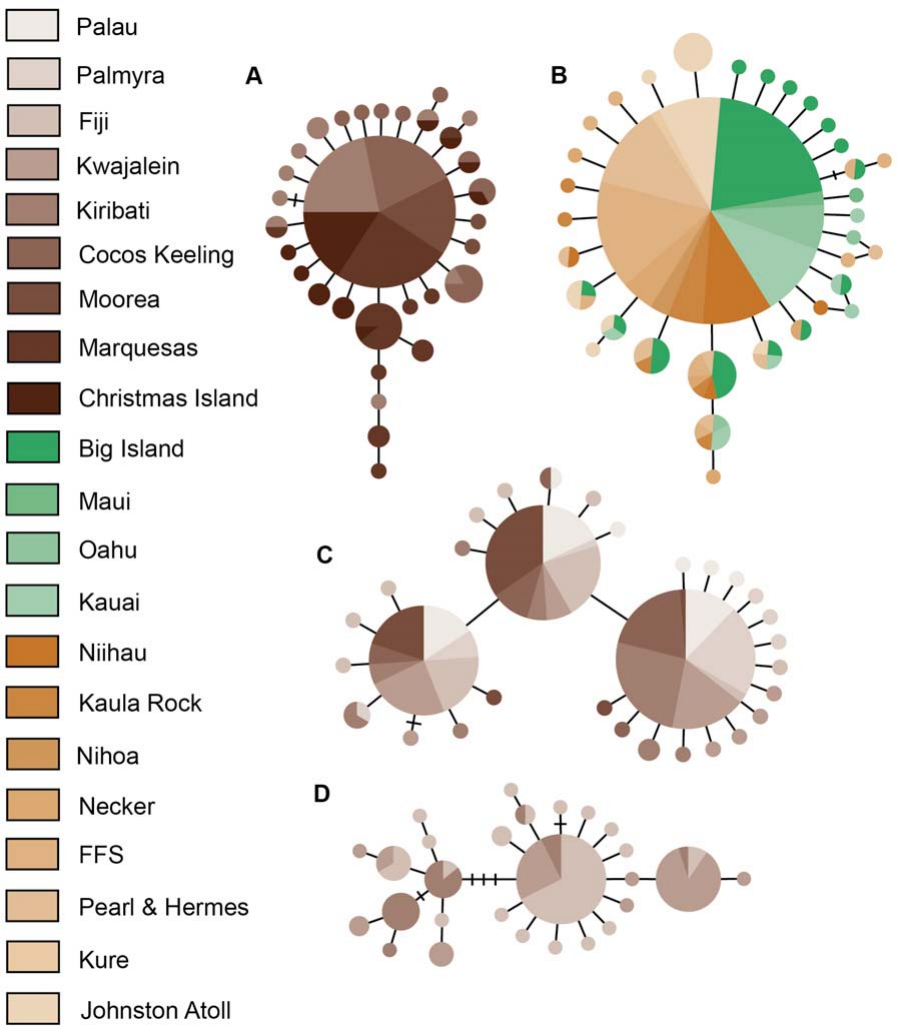
Cytochrome Oxidase 1 haplotype networks for all species. Minimum spanning networks showing CO1 haplotype relationships for A) *Halichoeres claudia* (slope), B) *H. ornatissimus* (slope), C) *H. trimaculatus* (lagoon), D) *H. margaritaceus* (lagoon). For *H. ornatissimus* the haplotypes are shown based on location, with shades of green colors representing the main Hawaiian Island and shades of orange colors representing the Northwest Hawaiian Islands. All other locations are represented by colors in the legend. Horizontal bars represent mutational steps between haplotypes. FFS  =  French Frigate Shoals.

**Figure 3:**
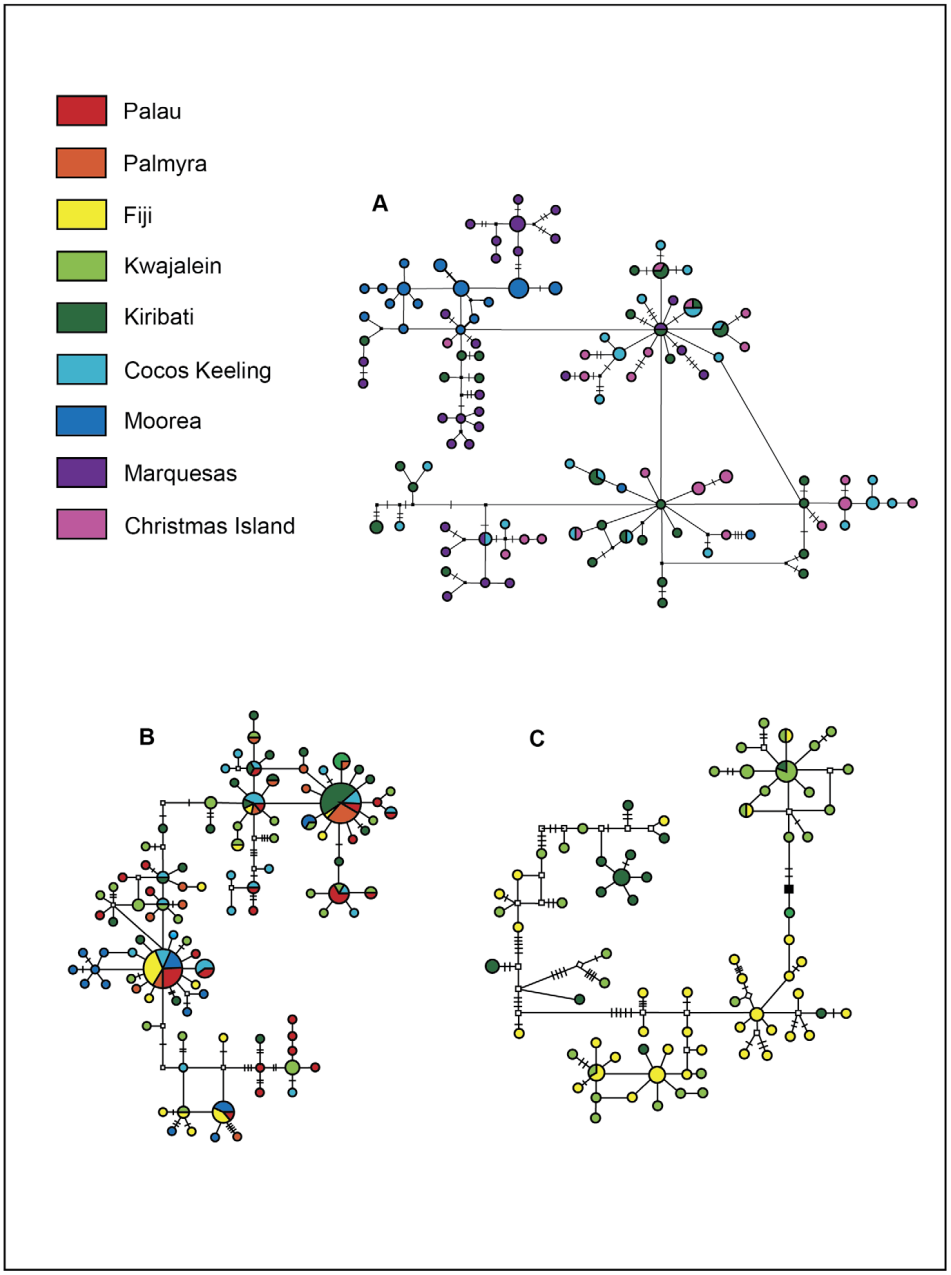
Control region haplotype networks for all species. Minimum spanning networks showing CR haplotype relationships for A) *Halichoeres claudia*, B) *H. trimaculatus*, C) *H. margaritaceus*. *H. ornatissimus* is not shown. Perpendicular bars and open squares represent single mutational steps between haplotypes, while closed squares represent 10 mutational steps.

**Figure 4:**
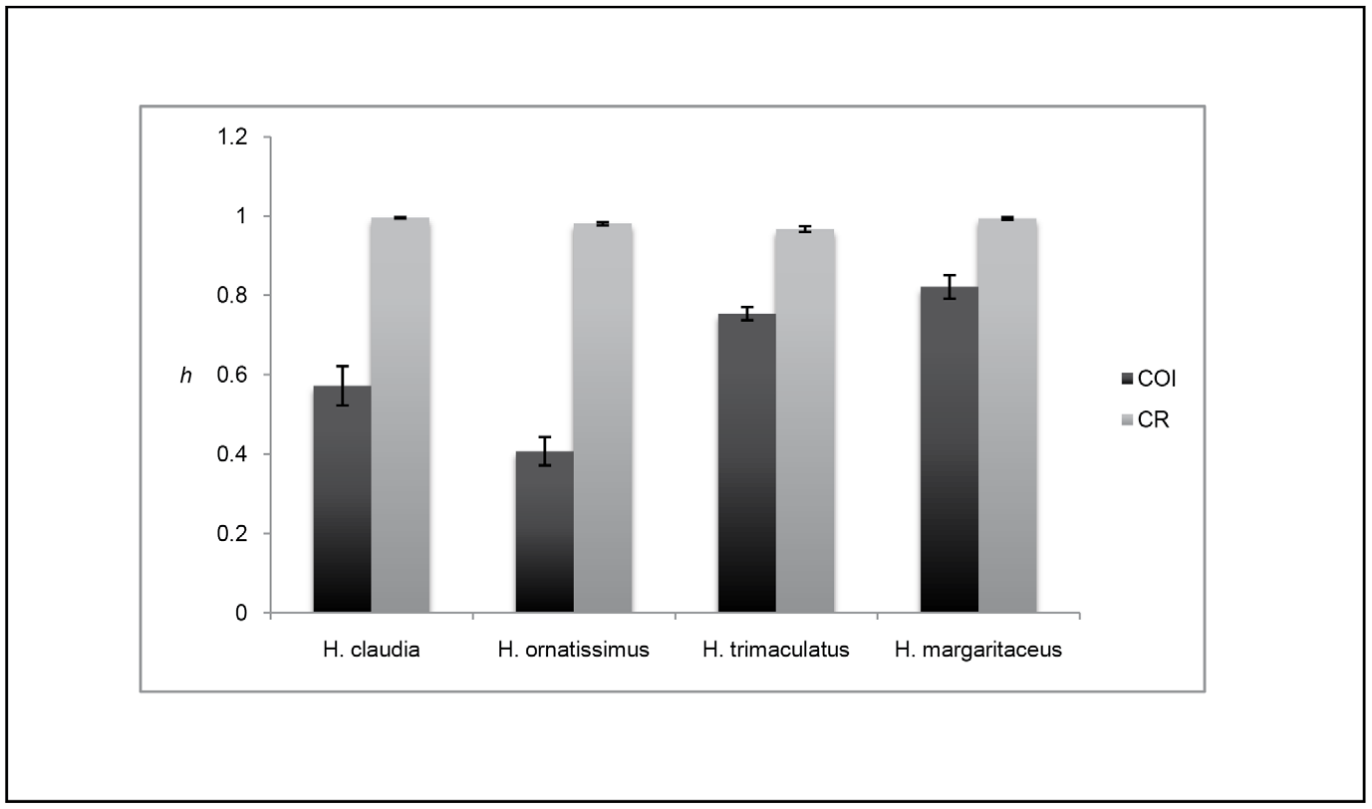
Haplotype diversities for each of four *Halichoeres* species. Haplotype diversities for each of four *Halichoeres* species with error bars. CO1 (dark grey) indicates diversities for the cytochrome oxidase subunit 1 fragment, CR (light grey) indicates diversities for the control region fragment.

A 526–559 bp segment of CO1, depending on the species, was obtained from 787 individuals, as well as a 252–343 bp segment of the CR from 723 individuals. Slope inhabitant *Halichoeres claudia* had 31 haplotypes for CO1 and 110 haplotypes for CR with 18 and 102 unique haplotypes (observed in single individuals), respectively (Fig. 2a & 3a). The minimum spanning network for CO1 shows a star pattern with the majority of individuals (65%) belonging to one haplotype (Fig. 2a). Overall haplotype diversity was 0.572±0.049 for CO1, and 0.996±0.002 for CR. Nucleotide diversity was 0.0018±0.0014 for CO1 and 0.0256±0.0135 for CR. Slope inhabitant *H. ornatissimus* was sampled at 12 locations across the Hawaiian archipelago, plus adjacent Johnston Atoll. CO1 had 34 haplotypes (21 unique), and CR had 162 haplotypes (122 unique; Fig. 2b). Similar to *H. claudia*, CO1 minimum spanning networks for *H. ornatissimus* show a star like pattern dominated by a single haplotype (Fig. 2b). Overall haplotype diversity was 0.407±0.036 for CO1 and 0.981±0.004 for CR. Nucleotide diversity was 0.0011±0.0010 for CO1 and 0.0268±0.0140 for CR. Lagoon inhabitant *H. trimaculatus* had 32 haplotypes for CO1 (26 unique), and 100 haplotypes for CR (78 unique; Fig. 2c & 3b). The CO1 minimum spanning network for *H. trimaculatus* shows three common haplotypes, each separated by one transition (Fig. 2c). Overall haplotype diversity was 0.754±0.017 for CO1 and 0.967±0.007 for CR. Nucleotide diversity was 0.0058±0.0033 for CO1 and 0.0268±0.0140 for CR. Lagoon inhabitant *H. margaritaceus* had 27 haplotypes for CO1 (18 unique), and 75 haplotypes for CR (66 unique; Fig. 2d & 3c). Overall haplotype diversity was 0.821±0.030 for CO1 and 0.994±0.003 for CR. Nucleotide diversities were 0.0022±0.0016 for CO1 and 0.0400±0.0200 for CR. Haplotype diversity and nucleotide diversities by sampling locations are provided in Tables 1 and 2 and a visual representation of overall haplotype diversity between species is provided in Figure 4. Overall haplotype and nucleotide diversity, Φ_ST_ estimates, and effective population sizes (N_E_) can be found in Table S1. Samples have been uploaded in GenBank with accession numbers JQ724865-JQ725555.

**Table 1:** Summary statistics for slope species.

Site	N	N_h_	*h*	*π*	Fu’s F
*H. Claudia*					
Marquesas	42, **32**	9, **30**	0.617±0.076, **0.994±0.011**	0.002±0.002, **0.025±0.014**	−3.49, **−25.06**
Moorea	18, **27**	3, **18**	0.216±0.124, **0.954±0.025**	0.000±0.001, **0.013±0.008**	−1.74, **−11.28**
Kiribati	31, **31**	10, **27**	0.546±0.108, **0.991±0.010**	0.002±0.001, **0.023±0.012**	−7.48, **−21.26**
Cocos-Keeling	32, **26**	8, **23**	0.595±0.094, **0.991±0.013**	0.001±0.001, **0.029±0.016**	−5.04, **−13.71**
Christmas Island	26, **23**	10, **21**	0.671±0.103, **0.992±0.015**	0.002±0.001, **0.023±0.013**	−8.64, **−15.19**
*H. ornatissimus*					
Big Island	71, **72**	16, **56**	0.502±0.073, **0.982±0.009**	0.001±0.001, **0.015±0.009**	−19.23, **−25.91**
Maui	6, **6**	2, **6**	0.333±0.215, **1.000±0.096**	0.001±0.001, **0.021**±**0.013**	0.00, **−2.03**
Oahu	17, **15**	3, **14**	0.228±0.130, **0.990±0.028**	0.001±0.001, **0.013±0.008**	−0.96, **−11.61**
Kauai	32, **29**	7, **28**	0.393±0.109, **0.998±0.010**	0.001±0.001, **0.020±0.011**	−4.55, **−25.51**
Kaula Rock	17, **20**	6, **18**	0.515±0.145, **0.984±0.024**	0.001±0.001, **0.015±0.008**	−3.77, **−15.37**
Niihau	27, **25**	4, **21**	0.214±0.103, **0.983±0.017**	0.000±0.001, **0.030±0.016**	−3.21, **−10.18**
Nihoa	7, **6**	1, **5**	0.000±0.000, **0.933±0.122**	0.000±0.000, **0.011±0.007**	0.00, **−1.46**
Necker	15, **19**	5, **16**	0.476±0.155, **0.983±0.022**	0.001±0.001, **0.014±0.008**	−2.17, −**11.64**
FFS	46, **46**	9, **33**	0.356±0.091, **0.965±0.018**	0.001±0.001, **0.014±0.008**	−6.74, **−26.05**
Pearl & Hermes	35, **37**	6, **28**	0.316±0.101, **0.978±0.014**	0.001±0.001, **0.014±0.008**	−4.09, **−25.42**
Kure	3,**4**	1, **4**	0.000±0.000, **1.000±0.177**	0.000±0.000, **0.019±0.014**	0.00, **−0.52**
Johnston Atoll	35, **30**	8, **20**	0.610±0.083, **0.949±0.024**	0.001±0.001, **0.014±0.008**	−4.77, **−12.65**

Summary statistics for slope species based on sample location. Control region values are in bold, and follow CO1 values. Number of individuals (N), number of haplotypes (N_h_), haplotype diversity (h), nucleotide diversity (π), and Fu’s F statistic are given for each location sampled. FFS  =  French Frigate Shoals.

**Table 2:** Summary statistics for lagoon species.

Site	N	N_h_	*h*	*π*	Fu’s F
*H. trimaculatus*					
Moorea	31, **20**	5, **14**	0.574±0.067, **0.947±0.034**	0.001±0.001, **0.021±0.012**	−1.37, **−4.87**
Palmyra	26, **19**	7, **13**	0.563±0.108, **0.906±0.060**	0.001±0.001, **0.021±0.012**	−3.73, **−3.82**
Kiribati	34, **31**	8, **19**	0.611±0.093, **0.875±0.056**	0.002±0.001, **0.017±0.010**	−3.19, **−9.01**
Kwajalein	35, **28**	8, **24**	0.726±0.050, **0.987±0.014**	0.002±0.001, **0.032±0.017**	−3.21, **−13.31**
Fiji	31, **22**	11, **13**	0.783±0.054, **0.866±0.066**	0.002±0.002, **0.018±0.010**	−7.17, **−3.74**
Palau	34, **33**	9, **24**	0.788±0.039, **0.958±0.023**	0.002±0.002, **0.031**±**0.017**	−3.98, **−9.88**
Cocos-Keeling	27, **24**	5, **18**	0.607±0.087, **0.967±0.023**	0.002±0.001, **0.029±0.016**	−0.77, **−6.31**
*H. margaritaceus*					
Kiribati	19, **17**	6, **14**	0.771±0.062, **0.971±0.032**	0.005±0.003, **0.030±0.016**	0.71, **−3.16**
Kwajalein	39, **36**	10, **32**	0.749±0.054, **0.989±0.012**	0.005±0.003, **0.041±0.021**	−1.03, **−15.06**
Fiji	50, **37**	19, **33**	0.706±0.072, **0.993±0.009**	0.004±0.002, **0.027±0.014**	−12.77, **−22.58**

Summary statistics for lagoon species based on sample location. Control region values are in bold, and follow CO1 values. Number of individuals (N), number of haplotypes (N_h_), haplotype diversity (h), nucleotide diversity (π), and Fu’s F statistic are given for each location sampled.

### Population Structure and Migration

For widespread slope species *H. claudia* 93% of the variance was within populations for CO1 and 87% for CR. All pairwise differences were significant for the Marquesas (Φ_ST_  = 0.074–0.183). In addition, CR showed significant structure for Moorea when compared to all other locations (Φ_ST_  = 0.183–0.251). For slope species *H. ornatissimus* (endemic to the Hawaiian Archipelago and adjacent Johnston Atoll) AMOVA revealed that 99% of genetic variation was within populations for both markers. None of the pairwise Φ_ST_ values were significant within the Hawaiian archipelago (*P*>0.05). When Hawaiian locations are compared to Johnston Atoll (1400 km southwest of Hawaii), most pairwise Φ_ST_ values were low but significant (Φ_ST_  = 0.001–0.097). For lagoon species *H. trimaculatus* AMOVA indicates 83–90% of the variation within populations, and for *H. margaritaceus*, AMOVA indicates 72–75% of the variation within populations. Lagoon species *H. trimaculatus* shows significant spatial structuring as well with Φ_ST_ values ranging from 0.019–0.495 (Table 3). For this species, Moorea and Fiji are significantly differentiated from all locations except each other. All six pairwise Φ_ST_ comparisons are significant for *H. margaritaceus* (Φ_ST_  = 0.155–0.419, Table 4). For both lagoon and slope species, all Mantel tests showed no significant relationship between pairwise Φ_ST_ and distance (r =  −0.349–0.269, *P*>0.424).

**Table 3:** Population pairwise Φ_ST_ values for *H. trimaculatus.*

*Sampling Location*	Palau	Kwajalein	Palmyra	Cocos	Kiribati	Moorea	Fiji
Palau	–	−0.013	0.050	0.004	0.124*	0.090*	0.071*
Kwajalein	0.021	–	0.030	−0.001	0.010*	0.084*	0.065*
Palmyra	0.149*	0.045	–	0.024	−0.012	0.224*	0.224*
Cocos	0.033	−0.007	0.016	–	0.080*	0.150*	0.137*
Kiribati	0.083*	0.019	−0.007	−0.013	–	0.335*	0.337*
Moorea	0.138*	0.300*	0.495*	0.342*	0.383*	–	−0.008
Fiji	0.063*	0.188*	0.365*	0.226*	0.278*	−0.004	–

Pairwise Φ_ST_ values for *H. trimaculatus* based on sampling location. Values for CO1 are given below the diagonal, while CR values are reported above.

* significant at *P* = 0.05.

**Table 4:** Population pairwise Φ_ST_ values for *H. margaritaceus.*

Sampling Location	Kiribati	Fiji	Kwajalein
Kiribati	–	0.304*	0.246*
Fiji	0.419*	–	0.208*
Kwajalein	0.305*	0.155*	–

Pairwise Φ_ST_ values for *H. margaritaceus* based on sampling location. Values for CO1 are given below the diagonal, while CR values are reported above.

* significant at *P* = 0.05.

Migrate results for each species did not converge on any specific point. This may be due to equal migration between all locations, or due to our study species not meeting assumptions of the software. Therefore our results from Migrate (not shown) are inconclusive.

### Neutrality and Population Expansion

**Figure 5:**
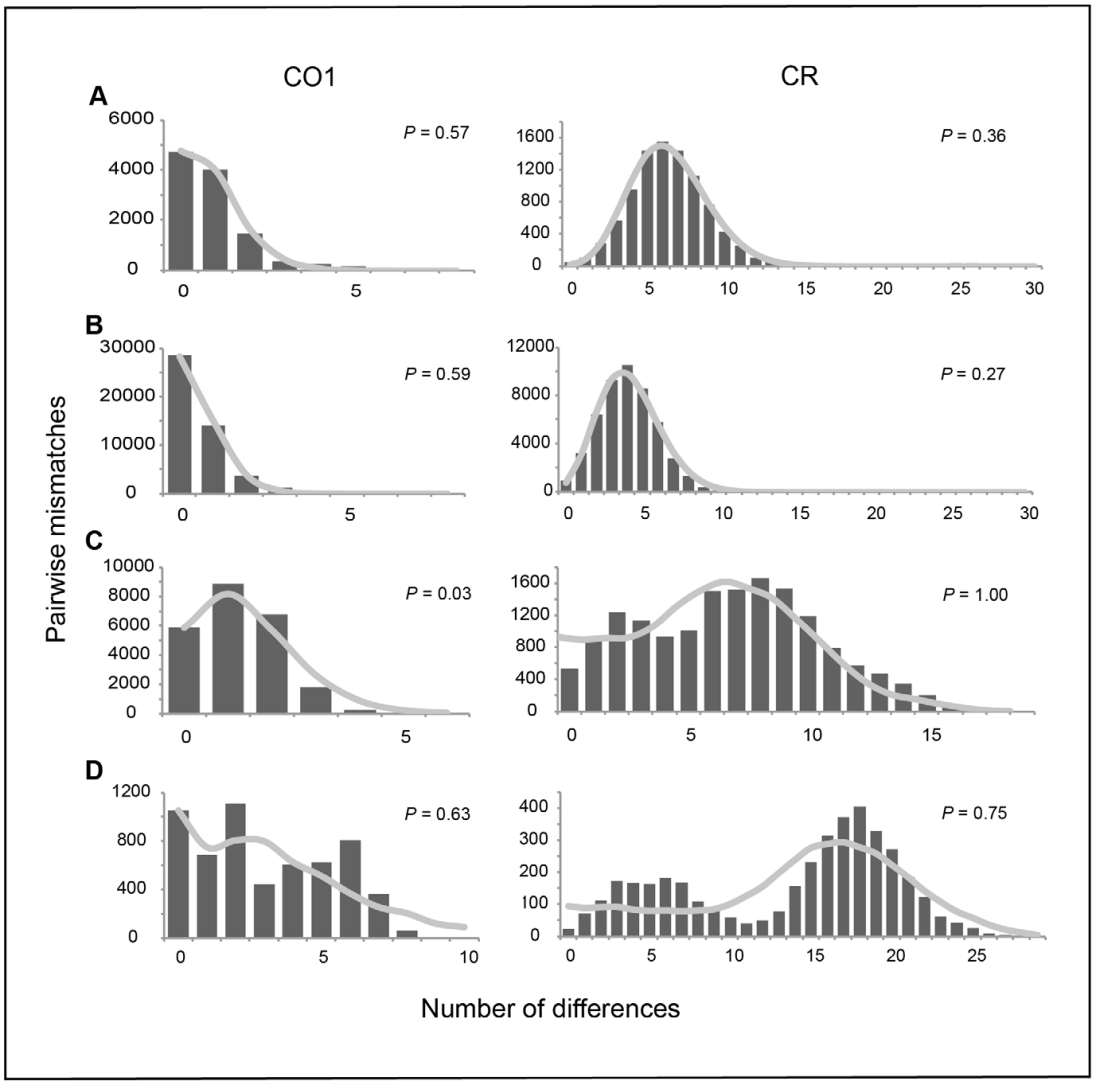
Simulated mismatch distributions for each species and molecular marker. Observed mismatch distributions are represented by the bar graphs, while the curve represents the simulated distribution. P-values are reported for each marker. A) *Halichoeres claudia*, B) *H. ornatissimus*, C) *H. trimaculatus*, D) *H. margaritaceus*.

All Fu’s F values were significant (*P*<0.001) and ranged from −31.71 to −14.55 (Tables 1 and 2). These values result from an excess of rare haplotypes, and indicate selection or population expansion [33]. Comparing the observed distribution of pairwise differences to simulated pairwise differences under a population expansion model [34], [35] failed to reject the model of sudden expansion in all species (*P*>0.13), except for CO1 in *H. trimaculatus* (*P* = 0.03, Fig. 5).

### Coalescent Estimates

Based on a 3% per million year mutation rate for CO1 and a 9% per million year mutation rate for CR, we were able to estimate time to most recent common ancestor (TMRCA) within each species. For CO1, slope species *H. claudia* and *H. ornatissimus* coalesce approximately 137,000 years before present (ybp; 95% CI 54,000−246,000 ybp) and 139,000 ybp (95% CI 37,000–299,000 ybp), respectively. For CR, the same species coalesce at approximately 277,000 ybp (95%CI 198,000−366,000 ybp), and 299,000 ybp (95% CI 220,000−385,000 ybp). These sister species are very similar and have only recently been described as distinct taxa [13], so we ran an additional BEAST analysis to determine the TMRCA including both species. CO1 coalesces at 223,000 ybp (95% CI 71,000–390,000 ybp), while CR coalesces to 346,000 ybp (95% CI 234,000–420,000 ybp). Lagoon species (*H. trimaculatus* and *H. margaritaceus*) coalesce to the late Pleistocene for both markers. *H. trimaculatus* coalesces at 350,000 ybp (95% CI 150,000–577,000 ybp) for CO1 and 484,000 ybp (95% CI 297,000–693,000 ybp) for CR. Similarly, *H. margaritaceus* coalesces to 107,000 ybp for CO1 (95% CI 34,000–211,000 ybp) and 399,000 ybp for CR (95% CI 210,000–616,000 ybp). We were able to estimate the rate of population increase for all species using the Bayesian skyline plot function in BEAST. Slope species show a more stable population through time (Fig. S1 A&B), while both lagoon species show a recent rapid increase in effective population size (Fig. S1 C&D). Even though most of the 95% confidence intervals overlap for both markers these are approximations which should be interpreted with caution. However, all values agree on a late Pleistocene (<500,000 yr) coalescence.

## Discussion

The survey of four wrasses with two mtDNA sequences was designed to assess connectivity and population history. Our expectation, based on previous research, was that lagoon wrasses would have shallow histories and perhaps more recent connections than species on the outer reef slope. Our results show the opposite; the lagoon wrasses show strong population structure and older population histories than their slope congeners. The range-wide study design allows us to make additional inferences about overall patterns of connectivity, and the generality of site-specific findings. Prior to dissecting these results, we mention four caveats relevant to this study:

1. *H. margaritaceus* was only collected at three locations due to either scarcity at sample locations or logistic limitations. As these three locations do not represent the species range in the central and West Pacific, results from *H. margaritaceus* should be regarded as provisional.
2. Clock rates for CO1 seem to cluster well among short-lived reef fishes (Table 3 in [29]) but control region rates are more controversial and probably more variable. With this uncertainty in mind, we caution against the over-interpretation of coalescence times. However, our rate estimate of 9%/MY is close to the estimate of 10%/MY used for marine angelfishes (Pomacanthidae) [26].
3. The second caveat leads to the observation that coalescence times from CR are two or three times higher than CO1 in three of the four species. For this reason we limit conclusions to a generic late Pleistocene timeframe rather than specific glacial events. The disparity between CR and CO1 results can be explained by several phenomena beyond the scope of this paper, but we note here that a mutation rate higher than our calibrations for CR, as has been suggested for other fish species (reviewed in [26]), would likely bring the two loci into closer alignment for coalescence times.
4. Our genetic analysis is based on two mtDNA loci that are linked due to the non-recombining nature of the vertebrate mitochondrial genome. Haploid inheritance makes mtDNA more sensitive to demographic events (including bottlenecks), and hence is appropriate to this study. However, the other edge of this sword is that species-wide natural selection on mtDNA (selective sweep [36]) could bias our findings. Although such events are rare [37], and we explicitly discount this possibility based on patterns of diversity (see Discussion), it is likely that investigations of the nuclear genome would be informative in resolving species histories.

### Population Expansion and Neutrality

Sea level variations due to Pleistocene glaciations have been used to explain genetic architecture in several reef fishes [6], [16], [17], [38]–[40]. The results of these studies are variable and lagoon/slope comparisons generally involved a single area or species. The goal of this study was to examine the effects of Pleistocene glaciations on lagoon and outer reef species across a large spatial scale. All species show relatively high haplotype diversity (*h*) and low nucleotide diversity (*π,* Fig. 4). This genetic signature can be attributed to rapid population expansion after a reduction in effective population size [41]. In support of this finding, Fu’s Fs values indicate a significant excess of rare haplotypes [33], [42], consistent with population expansion, although this can be subject to other interpretations (see below). All mismatch distributions, except for CO1 of *H. trimaculatus*, failed to reject a simulated population expansion model, providing additional evidence for a population expansion in all four species. Additionally, all skyline plots show an increase in effective population sized for all species, albeit at different rates (Fig. S1). These results are similar to previous studies on coral reef fish that have shown temporal, rather than geographic structuring between populations [6], [16], [17], [38], [39]. Coalescence times are consistent with population expansions associated with Pleistocene interglacial epochs, although not the most recent glacial maxima.

The finding of similar levels of genetic diversity (Tables 1 & 2, Fig. 4) was unanticipated, as we expected lagoon species to have low haplotype and nucleotide diversities consistent with fluctuating population sizes [6], [41]. Contrary to our expectations, lagoon species either exhibited similar (CR data) or greater (CO1 data) haplotype diversity than outer shelf species (Fig. 4). CR values are near saturation (approaching *h* = 1), so we place greater emphasis on genetic diversity estimates obtained from CO1 data. There are several possible scenarios that can explain these observations.

### High Genetic Diversity in Lagoon Species

Previous studies of genetic diversity in South Pacific reef fishes found evidence of population bottlenecks in both lagoon and outer reef species, but generally lower genetic diversity in lagoon species [6], [16]. These studies concluded that population bottlenecks were possibly due to reduced reef area, a higher degree of habitat disturbance, and possibly complete extirpation from some areas during low sea level [6]. The results of our study are not consistent with the previous studies in that the lagoon species examined here exhibit higher genetic diversity than outer reef species (Fig. 4).

The CO1 networks for lagoon species *H. trimaculatus* contain three haplotype clusters (Fig. 2c), but even though a strong network structure is evident, a phylogeographic signal is lacking as each of these clusters contains representatives from each location. This pattern is consistent with previous findings where species ranges may have been sundered during sea level lows and then subsequently reconnected [8], [39], [40]. Although the pattern is not as distinct with lagoon species *H. margaritaceus*, its CO1 haplotype network shows several clusters anchored by a common haplotype (Fig. 2d). Isolated refuges may have existed for lagoon species during sea level lows, depending on the bathymetry of their geographic range, which then could have mixed as they re-colonized habitats during sea level rises. This would result in the non-geographically structured haplotype networks observed in this study. Furthermore, if these refugia were isolated long enough for genetic differences to occur between populations, the mixing of these populations as sea levels rose again could inflate the genetic diversity compared to species that did not have multiple isolated populations during sea level lows, and result in haplotype networks similar to *H. trimaculatus* with multiple common haplotypes.

It is also possible that with a lack of lagoon habitat these species may survive in small patches of sandy, low energy habitat, even without a lagoon. These species have been observed outside of lagoons in sandy areas protected from high wave energy. Both lagoon species show bimodal mismatch distributions in the CR, which can have several interpretations. Given that contemporary effective population size estimates (N_E_s) are uniformly large (Table S1), and that lagoon and outer reef habitats are both abundant, current demographic processes are not likely influencing the different signatures between species. Primarily this bimodal signature is associated with fluctuating population sizes which is certainly possible given the multiple sea level fluctuations during the Pleistocene. Skyline plots indicated a rapid increase in population sizes for lagoon species (Fig. S1 C&D). However, this signature can also be interpreted as a signal of isolated populations that have recently come back into contact [43], which supports the concept of isolated refugia. Either interpretation leads us to hypothesize that lagoon wrasses have survived either in currently deep lagoons, wide continental margins, or the lee (protected) coastlines of oceanic islands during sea level lows [44].

### Outer Reef Species Diversity

In addition to the unexpected high levels of mtDNA diversity in lagoon species, another unexpected result of this study was the incongruence between markers for the outer reef species. Control region diversity levels were nearly saturated making interspecific comparisons difficult, while the CO1 region showed lower haplotype diversity for both *H. claudia* and *H. ornatissimus*. These results can be interpreted several different ways. A negative Fu’s F statistic [33] can indicate selection or population expansion. CO1 is a protein coding region of the mitochondrial DNA, whereas CR is a non-coding region, but (as noted above) these loci are linked and a selective sweep would affect both. The lack of this signal in both markers leads us to conclude that selection is not defining the mtDNA findings in these species.

Another possible explanation is that these outer reef species experienced a population bottleneck, indicated by the negative Fu’s F values and the star pattern of the minimum spanning network, and then underwent a spatial expansion. Previous studies have employed both temporal and spatial factors to describe genetic diversity patterns [38]. A spatial expansion following Pleistocene glaciations therefore may not have been observed in the CR data due to a mutation rate which is approximately three to four times higher than CO1. A recent spatial expansion could also explain the isolation by distance mantel tests, which were not significant for the outer reef species (r = −0.349–0.027, p>0.424). However, a large population expansion was not observed in skyline plots for outer reef species (Fig. S1 A&B). One other possible explanation is that populations of outer reef species have exchanged migrants consistently throughout the Pleistocene. This would explain the non-significant isolation by distance mantel tests, and the large amount of shared haplotypes in the CO1 networks (Fig. 2 A&B).

Slope species *H. claudia* and *H. ornatissimus* have a relatively long pelagic larval duration of approximately 40 days [45] that may allow greater dispersal and rapid colonization of new habitats. The lagoon species in this study have a shorter PLD (*H. margaritaceus* 21.7 days, *H. trimaculatus* 26.8 days) [45]. This may explain why *H. ornatissimus* is the sole representative of the genus in the Hawaiian archipelago. This would also explain why Indian Ocean populations are not distinct from Pacific populations in *H. claudia*, a pattern observed in other fish species with high dispersal ability [43], [46]–[49]. Although a meta-analysis reveals that the relationship between PLD and population genetic structure is not as simple as intuition would indicate [50], it is interesting that the lagoon species with shorter PLD are more genetically structured, consistent with earlier theories of reef fish connectivity [51].

Given this genetic evidence for high dispersal ability, we suggest that both *H. ornatissimus* and *H. claudia* have maintained connected populations throughout the Pleistocene, while experiencing population expansions across their ranges, as indicated by the CO1 haplotype networks (Fig. 2). This signature of population expansion likely occurred after a population bottleneck associated with Pleistocene glaciations, although on a reduced scale compared to lagoon species (Fig. S1). Reduced effective population sizes during the Pleistocene would result in the lower genetic diversity indices observed in these slope species.

### Conclusions

Sea level fluctuations during the Pleistocene rearranged reef habitat and severely altered lagoon habitat. Reduced habitat area may result in a reduced genetic diversity for lagoon species in comparison to outer reef species, at specific sites where lagoon species may have been extirpated. However, this study found no significant difference in genetic diversity between lagoon species and outer reef species, while examining the question at a large spatial scale. All species seem to have been affected by population bottlenecks during the Pleistocene, although at different intervals. During sea level lows it is possible that multiple refugia and lower dispersal allowed some populations of lagoon species to become isolated from one another either on continental margins or remote island chains, and subsequently reconnect, which resulted in the observed high genetic diversity and strongly temporally structured haplotype networks. This conclusion is supported by bimodal mismatch distributions and observed non-geographical patterns in the haplotype networks for lagoon species. The contrasting pattern for outer reef species can be explained by population expansion and a long pelagic larval duration that helped maintain connectivity between populations. Finally, we cannot rule out that the idiosyncrasies of individual species may explain some of the results [52], and acknowledge that only using mitochondrial markers limits the conclusions which can be drawn from this study. Ultimately the magnitude of the effects of Pleistocene glaciations most likely will be specific and unique based on each species particular life history, ecological, and demographic attributes.

## Supporting Information

Figure S1 **Simulated Bayesian skyline plots for each species and molecular marker.** Effective population sizes were estimated using Bayesian skyline plots in BEAST. The thick solid line in the middle of each figure represents the median estimates, while the thinner upper and lower line represent the 95% confidence intervals. The CO1 data is on the left, control region data on the right. A) *Halichoeres claudia*, B) *H. ornatissimus*, C) *H. trimaculatus*, D) *H. margaritaceus*.
(TIF)

Table S1 **Population summary statistics for each species.** Overall population statistics for each species. Control region values are in bold, following CO1 values. Φ_ST_, Haplotype diversity (*h*), nucleotide diversity (*π*), and effective population sizes (N_E_) are given for each species.
(DOCX)
