# Supplementary figures and images for: Living in the Past: Phylogeography and Population Histories of Indo-Pacific Wrasses (Genus Halichoeres) in Shallow Lagoons versus Outer Reef Slopes
Source: PLoS One. 2012 Jun 6;7(6):e38042. doi: 10.1371/journal.pone.0038042 (PMC3368945; doi:10.1371/journal.pone.0038042)

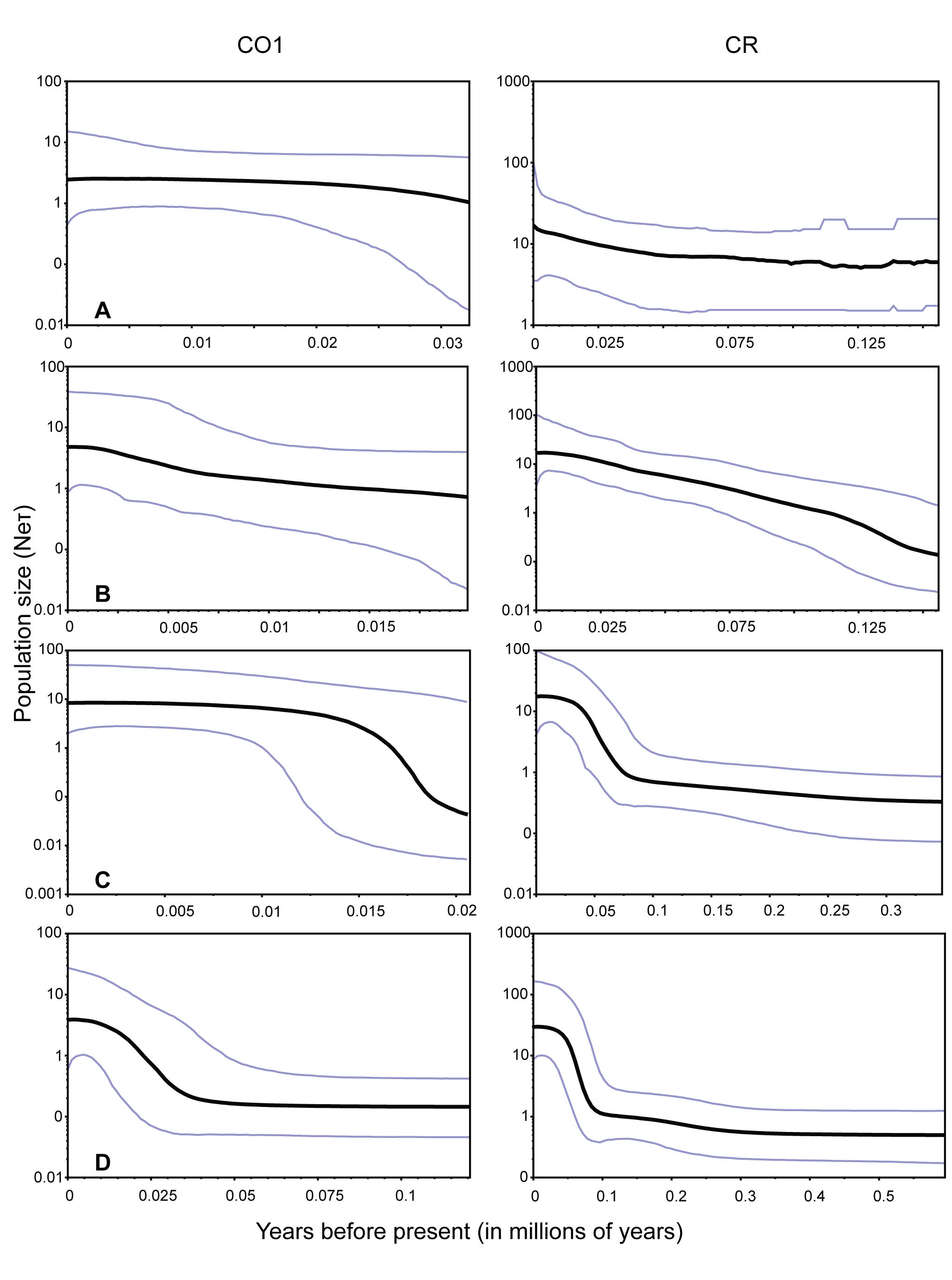

Supplement: Figure S1 — Simulated Bayesian skyline plots for each species and molecular marker. Effective population sizes were estimated using Bayesian skyline plots in BEAST. The thick solid line in the middle of each figure represents the median estimates, while the thinner upper and lower line represent the 95% confidence intervals. The CO1 data is on the left, control region data on the right. A) Halichoeres claudia, B) H. ornatissimus, C) H. trimaculatus, D) H. margaritaceus. (TIF) [file pone.0038042.s001.tif]
