# Supplementary material for: Living in the Past: Phylogeography and Population Histories of Indo-Pacific Wrasses (Genus Halichoeres) in Shallow Lagoons versus Outer Reef Slopes
Source: PLoS One. 2012 Jun 6;7(6):e38042. doi: 10.1371/journal.pone.0038042 (PMC3368945; doi:10.1371/journal.pone.0038042)
Supplement: Table S1 — Population summary statistics for each species. Overall population statistics for each species. Control region values are in bold, following CO1 values. ΦST, Haplotype diversity (h), nucleotide diversity (π), and effective population sizes (NE) are given for each species. (DOCX) [file pone.0038042.s002.docx]

Supporting Table 1. Population summary statistics for each species.

| Species | Φ_ST_ | *h* | *π* | N_E_ |
| --- | --- | --- | --- | --- |
| *H. claudia* | 0.064, **0.123** | 0.572 + 0.049, **0.996 + 0.002** | 0.002 + 0.001, **0.026 + 0.014** | 29,231; **141,790** |
| *H. ornatissimus* | 0.005, **0.009** | 0.407 + 0.036, **0.981 + 0.004** | 0.001 + 0.001, **0.016 + 0.009** | 18,015; **91,196** |
| *H. trimaculatus* | 0.166, **0.095** | 0.754 + 0.017, **0.967 + 0.007** | 0.002 + 0.002, **0.027 + 0.014** | 36,503; **148,634** |
| *H. margaritaceus* | 0.271, **0.241** | 0.821 + 0.030, **0.994 + 0.003** | 0.006 + 0.003, **0.040 + 0.020** | 96,021; **221,791** |

Overall population statistics for each species. Control region values are in bold, following CO1 values. Φ_ST_, Haplotype diversity (*h*), nucleotide diversity (*π*), and effective population sizes (N_E_) are given for each species.
